# Antenatal screening timeline and cutoff scores of the Edinburgh Postnatal Depression Scale for predicting postpartum depressive symptoms in healthy women: a prospective cohort study

**DOI:** 10.1186/s12884-022-04740-w

**Published:** 2022-06-28

**Authors:** Akiko Tanuma-Takahashi, Tomohiro Tanemoto, Chie Nagata, Ryo Yokomizo, Akiko Konishi, Kenji Takehara, Tetsuo Ishikawa, Nozomu Yanaihara, Osamu Samura, Aikou Okamoto

**Affiliations:** 1 Department of Obstetrics and Gynecology, The Jikei University School of Medicine, Nishi-Shinbashi 3-25-8, Minato-ku, Tokyo, 105-8461 Japan; 2 Center for Postgraduate Education and Training, National Center for Child Health and Development, 2-10-1 Okura, Setagaya-ku, Tokyo, 157-8535 Japan; 3 Department of Health Policy, National Center for Child Health and Development, 2-10-1 Okura, Setagaya-ku, Tokyo, 157-8535 Japan; 4 Advanced Data Science Project, RIKEN Information R&D and Strategy Headquarters, 1-7-22 Suehiro-cho, Tsurumi-ku, Yokohama, Kanagawa 230-0045 Japan

**Keywords:** Edinburgh Postnatal Depression Scale, Postpartum depression, Postpartum depressive symptoms, Screening, Cutoff, Prediction

## Abstract

**Background:**

It is worthwhile to identify women at risk of developing postpartum depression during pregnancy. This study aimed to determine the optimal time and cutoff score for antenatal screening for prediction of postpartum depressive symptoms (PDS) using the Edinburgh Postnatal Depression Scale (EPDS) and to identify risk factors for PDS.

**Methods:**

The target population was healthy pregnant women receiving antenatal care at a university hospital in Tokyo, Japan. During the first, second, and third trimesters, 3–4 days postpartum, and one month postpartum, they were asked to take the Japanese version of the EPDS questionnaire. The primary outcome of the study was PDS, defined as an EPDS score ≥ 9 at one month postpartum. The area under the receiver operating characteristics curve (AUC), sensitivity, specificity, positive predictive value (PPV), and negative predictive value (NPV) of EPDS scores at each antenatal screening time were calculated.

**Results:**

From 139 pregnant women, 129 were successfully followed up throughout the study. The number of women with an EPDS score ≥ 9 during the first, second, and third trimesters, 3–4 days postpartum, and one month postpartum were 6/126 (4.8%), 9/124 (7.3%), 5/117 (4.3%), 17/123 (13.8%), and 15/123 (12.2%), respectively. Screening during the second trimester had the highest AUC to predict PDS (0.89) among antenatal screenings. The optimal EPDS cutoff score during the second trimester was 4/5 (sensitivity: 85.7%; specificity: 77.1%; PPV: 33.3%; NPV: 97.6%). An EPDS score ≥ 5 during the second trimester (adjusted odds ratio [aOR]: 15.9; 95% confidence interval [95%CI]: 3.2–78.1) and a family history of mental illness (aOR: 4.5; 95%CI: 1.2–17.5) were significantly associated with PDS.

**Conclusions:**

Our study suggests that the EPDS score at the second trimester with the cutoff value of 4/5 may be adequate for initial screening for prediction of PDS. Women with an EPDS score ≥ 5 at the second trimester require more elaborate follow-up.

## Background

Postpartum depression (PPD) is one of the major health problems in peripartum women and has been reported to affect attachment to the infant, malnutrition in the infant by maternal inadequacy for childcare, and their subsequent cognitive and physical development [1]. According to a systematic review and meta-analysis which covered studies from multiple countries, even among healthy mothers without a prior history of depression, the incidence rate of PPD was 12% [2]. In Japan, the incidence rate of PPD was reportedly 15.1% within the first month and 11.6% during 1–3 months postpartum [3].

Previous studies have shown a correlation of PPD with socioeconomic problems (e.g., economic status, educational level of women, poor marital relationship, stressful life events, and lack of social support) [4–7], as well as obstetric complications and newborn conditions [8, 9]. Mental illness during and/or before pregnancy, particularly previous depression, is also an independent antenatal predictor of PPD [4].

The Edinburgh Postnatal Depression Scale (EPDS) is the most widely used screening tool for PPD [10, 11]. The EPDS screening was reported to be effective for detecting both antepartum and postpartum depression [12–16]. Postpartum depressive symptoms (PDS) have been defined as having a high EPDS score [4, 17, 18]. Some studies have investigated the predictive validity of the antenatal EPDS for predicting PDS [17–19].

It is worthwhile to identify women at risk of developing PDS before delivery as this allows medical professionals to prepare for and provide necessary medical services for those women in a timely manner. However, the optimal time for screening during pregnancy and the optimal cutoff score of the EPDS for prediction of PDS have not yet been established. The primary aim of the present study was to determine the optimal time for antenatal EPDS screening and the EPDS cutoff score for prediction of PDS at one month postpartum.

## Methods

### Target population and study sample

The present study was conducted as a part of a prospective cohort study which aimed to examine biological stress markers during pregnancy and their association with PPD. The target population of the study was healthy pregnant women who were receiving antenatal care at the Jikei University Hospital, which is a tertiary hospital located in central Tokyo and has approximately 800–900 deliveries per year. Between July 2014 and June 2015, pregnant women at 7–9 weeks of gestation were invited to participate in this study. The inclusion criteria were as follows: 1) healthy women (defined as women without any disease [internal diseases, mental disorders, or gynecological diseases] at diagnosis of the current pregnancy), 2) with fetal heartbeat confirmed by ultrasound, 3) receiving antenatal care at our institution from the first trimester, 4) planning to give birth at our institution, and 5) being able to answer the questionnaire written in Japanese. The exclusion criteria were women with coexisting complications (e.g., diabetes mellitus, thyroid diseases, hypertension), women with multiple pregnancies, and women who planned to deliver at other hospitals. It was pre-planned that women who miscarried or aborted at the first trimester and women who moved to other hospitals would be excluded from the analysis.

### Study schedule and measurements

As regular antenatal care, the following information was collected from all the participants at the first visit (maternal age, parity, mode of conception, complications in their previous pregnancies, education, past medical history, smoking, and alcohol consumption) and during the second and third trimesters (lifestyle, jobs, physical and mental condition, and expected support from their family). In addition to the regular antenatal care, participants were requested to respond to the questionnaires at 8–10 weeks of gestation (first trimester), at 24–26 weeks of gestation (second trimester), at 35–36 weeks of gestation (third trimester), at 3–4 days postpartum, and at one month postpartum. Each questionnaire included the Japanese version of EPDS and questions about sleeping hours, working hours, exercise habits, and support from their husbands/partners. There have been several reports which suggested that the EPDS during early postnatal days could be a useful screening instrument for early-onset PPD [5, 20]. Therefore, we included EPDS 3–4 days postpartum in the present study.

The EPDS is a 10-item self-reporting screening tool for PPD with each item scored on a 4-point scale ranging from 0 to 3, and total scores ranging from 0 to 30. The original English version of EPDS has acceptable identical consistency and reliability [10], and the Japanese version was confirmed to be equivalent to the original English version by Okano and others [21] with acceptable internal consistency and test–retest reliability [11].

### Statistical analysis

The primary outcome of the present study was PDS. In this study, we defined PDS as having a high EPDS (≥ 9) score at one month postpartum, which is considered to indicate a higher risk for PPD. The cutoff score of 8/9 at one month postpartum has been confirmed to be the most appropriate value for Japanese women (sensitivity 75%; specificity 93%) [21] and has been widely used to assess the risk of PPD in Japan [20, 22–26].

First, we investigated the trends in EPDS scores during pregnancy and postpartum. The EPDS screening during pregnancy was performed with the aim of predicting PDS, whereas the postpartum EPDS was performed with the aim of assessing PDS at that time. In order to determine the optimal screening time and cutoff score, we developed receiver operating characteristic (ROC) curves for each trimester by plotting the sensitivity against the “1 – specificity” of each cutoff value and calculated the area under the ROC curve (AUC). A value of 0.7–0.8 indicates a reasonable predictive accuracy, 0.8–0.9, a satisfactory accuracy, and a value of 0.9 or above is interpreted as excellent [27]. The difference among the AUCs obtained from EPDS scores at the first, second, and third trimester was tested using the Stata’s roccomp command [28]. The optimal cutoff score was determined using the Youden index, which is one of the statistical methods to obtain the best cutoff value for continuous variables [29].

Second, to investigate antenatal and perinatal risk factors for PDS, demographic, social, psychological, and physical factors were assessed in the preliminary univariate analysis. We tested whether each categorical variable was associated with PDS using Fisher’s exact test. We further conducted a multivariate logistic regression analysis. The main predictor in the multivariate logistic regression model was the variable created based on the results of the potential optimal screening time and cutoff score during pregnancy for prediction of PDS. In addition, variables with *P* values less than 0.05 in the univariate analysis were considered to be potential risk factors and were included in the multivariate analysis. Adjusted odds ratios (aOR) and corresponding 95% confidence intervals (CIs) were calculated. The participants with missing data were excluded from each analysis.

All statistical analysis was performed using Stata 14.0 (StataCorp LP, College Station, Texas, USA). *P* values less than 0.05 were considered to be statistically significant.

## Results

**Fig. 1:**
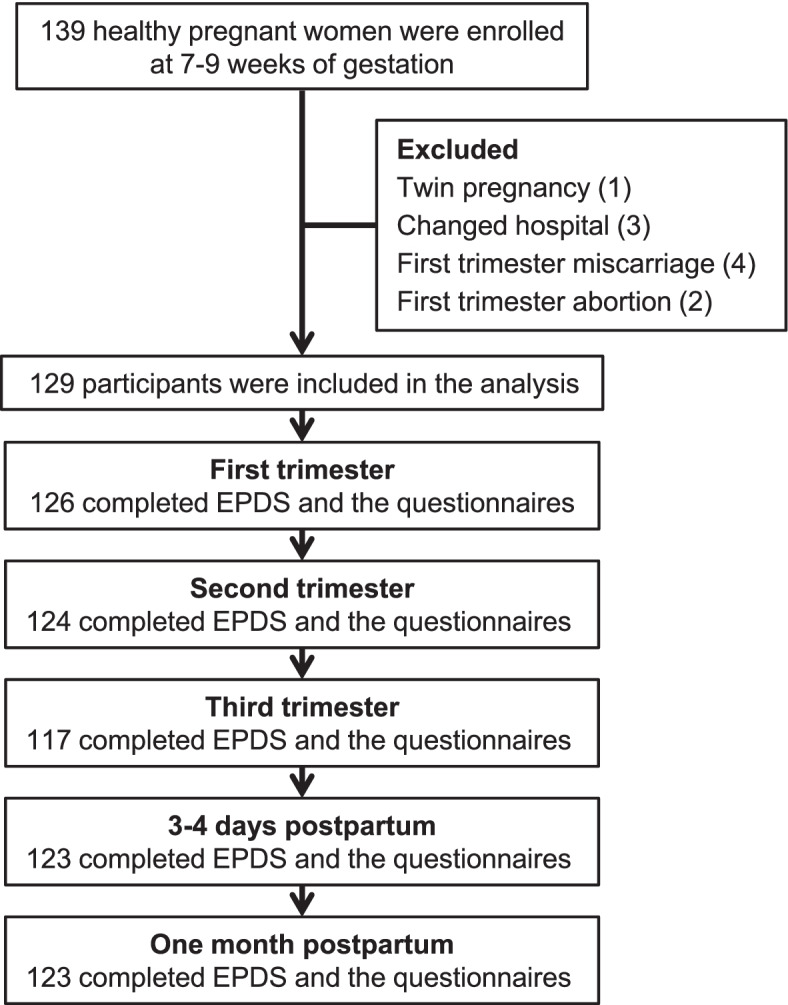
Flowchart of study participants

During the recruitment period, a total of 139 pregnant women were enrolled in the study. Out of 139 participants, there were 10 participants who dropped out (one for twin pregnancy, four and two for first trimester miscarriage and abortion, and three for hospital transfer). As a result, data from 129 participants were included in the analysis (Fig. 1). Of these, 126, 124, 117, 123, and 123 participants completed the questionnaires in the first, second, and third trimesters, at 3–4 days postpartum, and one month postpartum, respectively (Fig. 1).

### Characteristics of participants

The demographic characteristics of the participants are presented in Table 1A. The average maternal age was 32.9 years (range 26–41). All participants were Japanese and married. The most common family type was a nuclear family (95.2%). The majority of the participants graduated from a university or graduate school and were working full-time or part-time until they entered maternity leave (72.8% and 77.3%, respectively). Twenty-one participants (16.7%) had a family history of mental illness.

**Table 1:** Basic characteristics of study population (*N* = 129)

**[A] Socio-demographic characteristics**		
Variable		N (%)
Maternal age, years	≥ 40	6 (4.7)
	35–39	39 (30.2)
	26–34	84 (65.1)
	Missing	0
Parity	Primiparous	78 (60.5)
	Multiparous	51 (39.5)
	Missing	0
Mode of conception	Natural or timed intercourse	117 (90.7)
	Artificial insemination	5 (3.9)
	In-vitro fertilization	7 (5.4)
	Missing	0
Education	University or graduate school	83 (72.8)
	Junior college or technical school	21 (18.4)
	High school	4 (3.5)
	Others	6 (5.3)
	Missing	15
Smoking before pregnancy	No	116 (90.6)
	Occasionally	12 (9.4)
	Missing	1
Alcohol before pregnancy	No	25 (19.7)
	Occasionally	91 (71.7)
	Almost every day	11 (8.6)
	Missing	2
Marital status	Yes	129 (100)
Family type	Nuclear family	120 (95.2)
	Extended family	6 (4.8)
	Missing	3
Family history of mental illness	Yes	21 (16.7)
	Missing	3
Exercise at second trimester	Yes	34 (26.6)
	Missing	1
Working at second trimester	Full-time or part-time	99 (77.3)
	Homemaker	29 (22.7)
	Missing	1
**[B] Pregnancy and delivery outcomes**		
Variable		N (%)
Gestational weeks at delivery	< 37	6 (4.7)
	37–41	123 (95.3)
	Missing	0
Final mode of delivery	Natural vaginal	90 (69.8)
	Vacuum or forceps delivery	14 (10.8)
	Cesarean section, planned/emergency	13/12 (10.1/9.3)
	Missing	0
Epidural anesthesia	Yes	46 (35.7)
	Missing	0
Complications during delivery	Weak pain or prolonged labor	22 (17.0)
	Non-reassuring fetal status	7 (5.4)
	Hypertensive disorders of pregnancy	5 (3.9)
	Atonic postpartum hemorrhage	5 (3.9)
	Fetal abnormality found postpartum	2 (1.6)
	Uterine infection	1 (0.8)
	Others	7 (5.4)
	Missing	0
Feeding	Breast-feeding	52 (42.3)
	Breast-feeding plus formula	69 (56.1)
	Formula	2 (1.6)
	Missing	6

Table 1B presents delivery outcomes of the participants. The average gestational age at delivery was 38.5 weeks (range 31–41). The modes of delivery were natural vaginal delivery (90, 69.8%), vacuum or forceps delivery (14, 10.8%), planned cesarean section (13, 10.1%), and emergency cesarean section (12, 9.3%). Forty-six (35.7%) participants requested and received labor analgesia. Forty-nine (38.0%) had delivery complications.

### Trends of EPDS scores

**Fig. 2:**
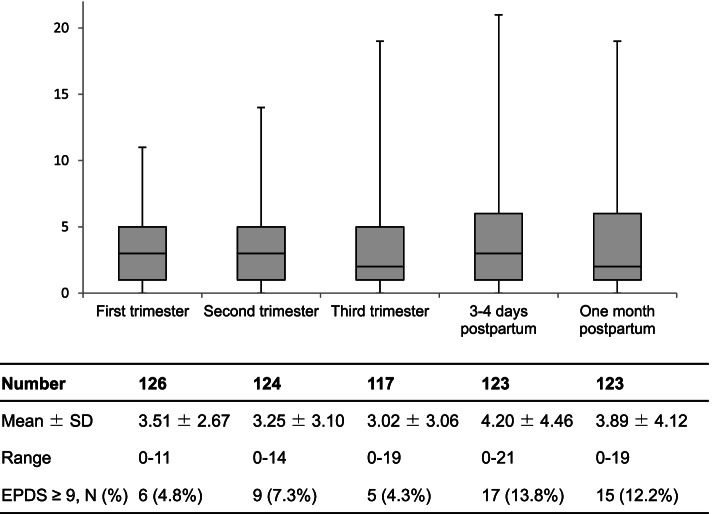
Trends of EPDS scores during pregnancy and postpartum period. SD, standard deviation

The trends of EPDS scores are shown in Fig. 2. The mean EPDS scores in the first, second, and third trimesters, at 3–4 days postpartum, and at one month postpartum were 3.51 (standard deviation [SD]: 2.67), 3.25 (SD: 3.10), 3.02 (SD: 3.06), 4.20 (SD: 4.46), and 3.89 (SD: 4.12), respectively. The mean EPDS score was highest at 3–4 days postpartum. On the other hand, the mean EPDS score was lowest in the third trimester.

### ROC curves for the prediction of PDS

**Fig. 3:**
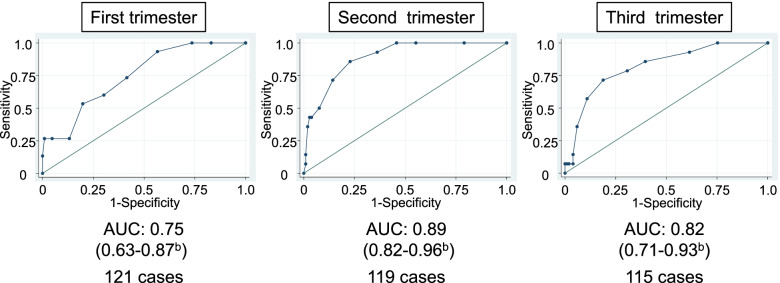
Receiver operating characteristic (ROC) curves and areas under the ROC curve (AUC) of Edinburgh Postnatal Depression Scale (EPDS) scores at first, second, and third trimester of pregnancy to predict postpartum depressive symptoms^a^. ^a^Postpartum depressive symptoms were defined as an EPDS score ≥ 9 at one month postpartum. ^b^ 95% confidence intervals. AUC, area under the curve

The ROC curve for each trimester was developed based on the data from participants who answered the questionnaire both at that trimester and one month postpartum (Fig. 3). The statistical test for the AUCs at the first, second, and third trimester revealed a statistically significant difference for predicting PDS among them (*P* = 0.01). The AUC at the second trimester was higher among the AUCs during the antenatal period (0.89, 95% CI: 0.82–0.96). Sensitivity, specificity, positive predictive value (PPV), and negative predictive value (NPV) for each of the possible cutoff scores of EPDS at the second trimester are shown in Table 2. A cutoff score of 3/4 had a quite high sensitivity (92.9%); however, the specificity was low (63.8%). On the other hand, cutoff scores of 5/6 and 6/7 had higher specificity (85.7%, 92.4%), whereas the sensitivity was low (71.4%, 50.0%). The Youden index indicated that the cutoff score of 4/5 was reasonable for predicting PDS (sensitivity: 85.7%; specificity: 77.1%; PPV: 33.3%; NPV: 97.6%).

**Table 2:** Predictive validity of each cutoff score of EPDS at second trimester for predicting postpartum depressive symptoms^a^

Cutoff score^b^	N^c^ (%)		Sensitivity, %	Specificity, %	PPV, %	NPV, %	PLR	NLR
EPDS ≥ 4		52 (42.7)	92.9	63.8	25.5	98.5	2.6	0.1
EPDS ≥ 5		37 (29.8)	85.7	77.1	33.3	97.6	3.8	0.2
EPDS ≥ 6		26 (21.0)	71.4	85.7	40.0	95.7	5.0	0.3
EPDS ≥ 7		16 (12.9)	50.0	92.4	46.7	93.3	6.6	0.5

*NLR* negative likelihood ratio, *NPV* negative predictive value, *PLR* positive likelihood ratio, *PPV* positive predictive value

^a^Postpartum depressive symptoms were defined as an EPDS score ≥ 9 at one month postpartum

^b^Cutoff score of EPDS at the second trimester

^c^This analysis included participants who completed EPDS both at the second trimester and one month postpartum (*N* = 124)

### Antenatal and perinatal risk factors for PDS

The number of women with an EPDS score ≥ 9 at one month postpartum was 15/123 (12.2%). Antenatal and perinatal risk factors for predicting PDS are summarized in Table 3. Family history of mental illness was the only statistically significant risk factor for PDS found in the univariate analysis.

**Table 3:** Univariate analysis of antenatal and perinatal risk factors for postpartum depressive symptoms^a^

Variable (N)	Postpartum depressive symptoms^a^		*P* value^b^
	Yes, (*N* = 15), N (%)	No, (*N* = 108), N (%)	
**[A] Antenatal risk factors**			
Maternal age, years			
< 35 (84)	13 (86.7)	66 (61.1)	0.082
≥ 35 (45)	2 (13.3)	42 (38.9)	
Primiparous (78)	11 (73.3)	62 (57.4)	0.276
Mode of conception			
Natural conception/Timed intercourse (117)	15 (100)	96 (88.9)	0.358
Artificial insemination/In-vitro fertilization (12)	0 (0)	12 (11.1)	
Education			
University or graduate school (83)	10 (66.7)	70 (64.8)	0.616
Junior college or technical school (21)	3 (20.0)	16 (14.8)	
High school (4)	1 (6.7)	3 (2.8)	
Others (6)	0 (0)	6 (5.6)	
Smoking before pregnancy			
No (116)	12 (80.0)	98 (90.7)	0.167
Yes (12)	3 (20.0)	9 (8.3)	
Alcohol before pregnancy			
No (25)	3 (20.0)	21 (19.4)	0.245
Occasionally (91)	9 (60.0)	77 (71.3)	
Almost every day (11)	3 (20.0)	8 (7.4)	
Family type			
Nuclear family (120)	14 (93.3)	100 (92.6)	0.559
Extended family (6)	1 (6.7)	5 (4.6)	
Family history of mental illness (21)	7 (46.7)	13 (12.0)	0.004
Regular exercise at second trimester (34)	7 (46.7)	25 (23.2)	0.064
Working at second trimester (99)	13 (86.7)	84 (77.8)	0.736
Family support			
No (5)	0 (0)	4 (3.7)	1
Yes (116)	13 (86.7)	98 (90.7)	
**[B] Perinatal risk factors**			
Preterm delivery, < 37 weeks (6)	0 (0)	4 (3.7)	1
Final mode of delivery			
Normal vaginal delivery (90)	15 (100.0)	72 (66.7)	0.102
Vacuum or forceps delivery (14)	0 (0)	13 (12.0)	
Cesarean section (25)	0 (0)	23 (21.3)	
Painless delivery (46)	7 (46.7)	37 (34.3)	0.395
Pregnancy and delivery complications, total (49)	2 (13.3)	43 (39.8)	0.050
Weak pain or prolonged labor (22)	0 (0)	22 (20.3)	
Non-reassuring fetal status (7)	0 (0)	7 (6.5)	
Hypertensive disorders of pregnancy (5)	1 (6.7)	3 (2.8)	
Atonic postpartum hemorrhage (5)	1 (6.7)	3 (2.8)	
Fetal abnormality found postpartum (2)	0 (0)	1 (0.9)	
Uterine infection (1)	0 (0)	0 (0)	
Others (7)	0 (0)	7 (6.5)	

^a^Postpartum depressive symptoms were defined as an EPDS score ≥ 9 at one month postpartum

^b^Analysis by Fisher’s exact test

### Multivariate regression models

The variable chosen as the main predictor was EPDS score ≥ 5 at the second trimester, which is a variable identified through the preceding analysis. Family history of mental illness, which had a *P* value of less than 0.05 in the univariate analysis, was included in the multivariate regression model. EPDS score ≥ 5 at the second trimester was a strong predictor of PDS. The aOR of developing PDS was 15.9 (95%CI: 3.2–78.1) for EPDS ≥ 5 at the second trimester by the multivariate logistic regression analysis. The aOR of developing PDS was 4.5 (95%CI: 1.2–17.5) for family history of mental illness.

## Discussion

In this study, we found that the predictive ability of antenatal EPDS for prediction of PDS were significantly different depending on when the screening was performed. The AUC of EPDS scores at the second trimester was higher for prediction of PDS. Regarding the EPDS score at the second trimester, the cutoff score of 4/5 seemed to be reasonable considering the balance between sensitivity and specificity.

### Screening timeline

Previous studies on the EPDS during the antepartum period generally aimed to validate the diagnostic accuracy of the EPDS for antepartum depression [14, 15, 30]. Other studies aimed to predict PDS using antenatal EPDS score; however, they obtained EPDS scores only once or twice during pregnancy at various screening times, or the EPDS was validated using only certain cutoff scores [4, 17, 19]. In our study, among the EPDS scores in the first, second, and third trimester, the EPDS score at the second trimester had the highest predictive ability for PDS. Generally, pregnant women have a more stable physical condition during the second trimester [31]; morning sickness has a non-negligible impact on women’s metal condition during the first trimester [32]; prenatal stress and physical discomfort (such as leg edema, labored breathing, and added weight) have a negative impact on health-related quality of life during the third trimester [33]. Thus, EPDS scores during the second trimester might reflect women’s mental condition more clearly without being affected by their physical condition.

### Cutoff score

There have been several studies which reported that the cutoff score of 4/5 could be used for the initial antenatal screening to detect women at risk of developing PDS, defined as postnatal EPDS score ≥ 10 [18, 19]. Our study obtained results similar to these studies. In their studies, as well as ours, this low cutoff score enabled reaching a high NPV over 96% [18, 19], which means that women scoring < 5 on the antenatal EPDS can be reassured that it is very unlikely that they will develop PDS.

### Risk factors

The antenatal EPDS is reported to perform better for prediction of PDS when combined with other predictors/risk factors, such as a prior history of major depression before pregnancy and low partner support [4, 19]. In our study, EPDS ≥ 5 at the second trimester and family history of mental illness were identified as risk factors for PDS. There was no significant relationship between PDS and other risk factors such as socioeconomic problems, obstetric factors, and newborn conditions in our analysis unlike other studies. This may be because of the difference in background characteristics of the study populations as discussed in the Strengths and Limitations section below.

### Strengths and limitations

The strength of our study is that EPDS scores were obtained at the first, second, and third trimesters of pregnancy and one month postpartum. This enabled us to investigate the optimal time for screening to identify women at risk of developing PDS.

Nonetheless, our study has some limitations. The participants of the present study were pregnant women who were seen at a university hospital located in central Tokyo, who generally had good educational attainment, high socioeconomic status, and familial support. In addition, we only enrolled healthy women without co-existing diseases. This resulted in relatively low frequency of PDS and may jeopardize the generalizability of our study findings.

## Conclusions

The EPDS score at the second trimester with the cutoff value of 4/5 may be adequate for initial screening for prediction of PDS. Women with an EPDS score ≥ 5 at the second trimester require more elaborate follow-up. Further research is needed to confirm this and better understand the risk factors for PPD in order to identify high-risk women during pregnancy.

## Data Availability

The datasets generated and analyzed during the current study are not publicly available following the relevant guidelines in Japan, but are available from the corresponding author on reasonable request.

## Abbreviations

aOR: Adjusted Odds Ratio
AUC: Area Under the ROC Curve
CI: Confidence Interval
EPDS: Edinburgh Postnatal Depression Scale
NLR: Negative Likelihood Ratio
NPV: Negative Predictive Value
PDS: Postpartum Depressive Symptoms
PLR: Positive Likelihood Ratio
PPD: Postpartum Depression
PPV: Positive Predictive Value
ROC: Receiver Operating Characteristic
SD: Standard Deviation
